# A Fast and Effective Spike Sorting Method Based on Multi-Frequency Composite Waveform Shapes

**DOI:** 10.3390/brainsci13081156

**Published:** 2023-08-02

**Authors:** Ruixue Wang, Yuchen Xu, Yiwei Zhang, Xiaoling Hu, Yue Li, Shaomin Zhang

**Affiliations:** 1 Qiushi Academy for Advanced Studies, Zhejiang University, Hangzhou 310027, China; 2 Department of Biomedical Engineering, Zhejiang University, Hangzhou 310027, China; 3 Institute of Advanced Study, Westlake Institute for Advanced Study, Hangzhou 310024, China; 4 CenBRAIN Neurotech, School of Engineering, Westlake University, Hangzhou 310030, China; 5 Department of Biomedical Engineering, The Hong Kong Polytechnic University, Hong Kong 100872, China; 6 Zhejiang Laboratory, Research Institute of Intelligent Computing, Hangzhou 311121, China; 7 Key Laboratory of Biomedical Engineering of Ministry of Education, Zhejiang University, Hangzhou 310027, China; 8 Zhejiang Provincial Key Laboratory of Cardio-Cerebral Vascular Detection Technology and Medicinal Effectiveness Appraisal, Zhejiang University, Hangzhou 310027, China

**Keywords:** spike sorting, high-pass filter, waveform, sorting accuracy

## Abstract

Accurate spike sorting to the appropriate neuron is crucial for neural activity analysis. To improve spike sorting performance, it is essential to fully leverage each processing step, including filtering, spike detection, feature extraction, and clustering. However, compared to the latter two steps that were widely studied and optimized, the filtering process was largely neglected. In this study, we proposed a fast and effective spike sorting method (MultiFq) based on multi-frequency composite waveform shapes acquired through an optimized filtering process. When combined with the classical PCA-Km spiking sorting algorithm, our proposed MultiFq significantly improved its sorting performance and achieved as high performance as the complex Wave-clus did in both the simulated and in vivo datasets. But, the combined method was about 10 times faster than Wave-clus (0.16 s vs. 2.06 s in simulated datasets; 0.46 s vs. 2.03 s in in vivo datasets). Furthermore, we demonstrated the compatibility of our MultiFq by combining it with other sorting algorithms, which consistently resulted in significant improvement in sorting accuracy with the maximum improvement at 35.04%. The above results demonstrated that our proposed method could significantly improve the sorting performance with low computation cost and good compatibility by leveraging the multi-frequency composite waveform shapes.

## 1. Introduction

Extracellular recording picks up electric potential fluctuations from the neurons surrounding the electrode tips along with background noises [1,2]. The recorded fluctuations indicate neural activities such as the action potentials (“spikes”) from a few unknown neurons at the targeted area. The recorded neural activities can then be used by neuroscientists to study brain functions. With the development of neuroscience and neurotechnology, the demand for neural activity analysis at the single neuron and neuron population level has dramatically increased in recent years. Accurate spike sorting, which assigns each detected spike waveform to the appropriate neuron, is crucial for subsequent research. However, spike sorting algorithms face challenges due to various waveform similarity and noise level.

Classical spike sorting frameworks consist of a series of processing, including filtering, detection, feature extraction, and clustering [3]. To improve the performance of spike sorting, it is essential to improve the performance of each processing step. Much effort has been devoted to the latter two steps to cluster spike waveform shapes in a suitable feature space [4,5]. The most common feature extraction is performing a principle component analysis (PCA) or choosing a wavelet basis [6,7]. Recent methods have combined the two to improve the sort performance [8]. In addition to the traditional clustering based on a mixture of Gaussians, a nonparametric clustering algorithm, such as superparamagnetic clustering (SPC), is another option [6]. The spike sorting toolbox Wave-clus, which combined the wavelet transform for feature extraction and SPC for clustering, is widely used. In addition, some modern algorithms even introduce template matching methods such as SpyKING Circus and KiloSort to improve the performance of feature extraction and clustering [9,10].

On the contrary, the filtering process, as the first step or pre-processing of spike sorting, has been largely neglected. The filtering process mentioned here usually refers to filtering raw neural data collected from the acquisition system with a digital high-band pass filter. After filtering, the spikes are detected with a pre-set threshold [3,4], and then the waveform shapes are extracted. Previous studies have confirmed that the spikes from a given neuron maintain the same shape over a period of time [11]. In most studies, the filtering process usually uses a high-pass filter with a cut-off frequency of 300–3000 Hz that is empirically chosen [12,13,14,15]. Even some new algorithms of spike sorting mainly for high-density microelectrode arrays, such as KiloSort and MountainSort, also adopt the empirical cut-off frequency at 300 Hz or 600 Hz [13,16]. However, it is noticed that the nonlinear phase responses of the most used causal infinite impulse response (IIR) bandpass filters introduce dramatic distortions to the signal of interest, including distorting the spike shapes themselves and changing the appearance of signal artifacts to make them indistinguishable from real spikes [17]. Despite the distortion in waveform shapes induced by the filtering process, most spike sorting algorithms have to rely on the distorted time-domain spike waveform shapes in the following two sorting steps. In addition to that, there are some applications for which the actual spike shapes are important. For example, the information is critical to distinguish the spikes between pyramidal and inhibitory neurons or study the relationship between intra- and extra-cellular action potentials [18,19].

Therefore, given that the filtered data could retain more information about the original spike waveform shapes or enhance differences between the spike waveform shapes from different units, the subsequent sorting steps would be benefited and simplified, and the whole sorting algorithm could be optimized. Some designed digital filters have been proposed to reduce the nonlinear phase responses in the filtering process, such as nearly-phase IIR filters [20,21] and zero-phase filters. However, the filter complexity and high time consumption made them inconducive to practical applications. Whether these optimized filters are beneficial to spike sorting performance remains to be further explored.

In this study, we proposed a spike sorting method (MultiFq) based on composite waveform shapes acquired through multiple high-pass filters. In the common filtering step, the obtained waveform shapes primarily lie in the time domain near the cut-off frequency band. Considering that other frequency bands could bring additional waveform features that are beneficial for the subsequent sorting processes, we filtered raw neural data with parallel and multiple high-pass filters with different cut-off frequencies and generated composite waveform shapes for the subsequent sorting processes. Our results demonstrated that our proposed method significantly improved the sorting performance with low computation cost and good compatibility.

## 2. Materials and Methods

As mentioned above, spike sorting methods commonly contain four steps: filtering, detection, feature extraction, and clustering. The previous strategies for the first two steps usually employ a single high-pass filter with a cut-off frequency of 300–3000 Hz, followed by detecting peaks and cutting waveforms [3,14]. Our MultiFq method introduced a novel approach by filtering the raw data with multiple high-pass filters with different cut-off frequencies. The waveforms obtained from different filters were integrated to generate composite features of spikes.

### 2.1. Theoretical Illustration

**Figure 1:**
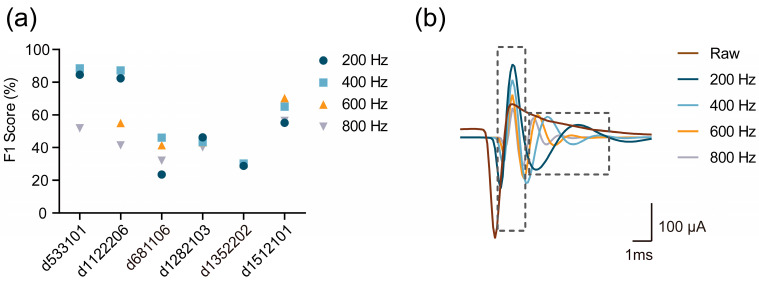
Theoretical illustration schematic. (**a**) The plot of the F1 score under different cut-off frequency on 6 testing sets in HC1 (see Section 2.6). (**b**) Waveform shapes under different filtering frequencies, 200 Hz, 400 Hz, 600 Hz, and 800 Hz, along with the raw waveform.

To demonstrate how the cut-off frequencies of high-pass filters influence spike sorting performance through the spike waveform shape, we selected four cut-off frequencies, 200 Hz, 400 Hz, 600 Hz, and 800 Hz for high-pass filtering, and evaluated the sorting performance (measured by F1 score, see Section 2.6) on six testing datasets in HC1 (see Section 2.6). Figure 1a illustrates the performance achieved with different cut-off frequencies. It is worth noting that the cut-off frequency of the highest performance varies across different testing datasets. In other words, the optimal cut-off frequency differs for different spike data and may not be necessarily equal to the commonly used range of 300–3000 Hz in practical applications. There are several possible reasons for this phenomenon:

Firstly, different frequency high-pass filters capture distinct aspects of waveform features due to shape distortion induced by filtering. Figure 1b shows the various waveform shapes obtained from the same raw waveform under different cut-off frequencies (200 Hz, 400 Hz, 600 Hz, and 800 Hz). The waveforms from lower filtering frequencies (200 Hz and 400 Hz) exhibit higher peak–peak values (P-P values), i.e., the P-P value of the waveform decreases as the cutoff frequency increases. We could deduce that lower frequency bands are more effective at capturing amplitude features during depolarization (Figure 1b, left grey dotted box). On the other hand, the waveforms from higher frequency band filters display more rugged tails (Figure 1b, right grey dotted box). Thus, higher frequency bands might be more effective at capturing more features during hyperpolarization.

Secondly, different frequency components resist noise with different power spectra. Our previous study found that higher frequency bands had stronger noise resistances when the noise conformed to 1/f distribution in the frequency domain [22]. However, when the noise structure deviated from the 1/f model, higher frequency bands were inferior to the lower ones. As a matter of fact, the power spectra of realistic noises in various in vivo recordings always deviated from the 1/f model. We investigated the noise structures in numerous in vivo recordings of different regions, including the motor cortex (M1), the hippocampus (CA1), the brainstem, and the visual cortex (V1), from rodents and non-human primates [23]. After analyzing how well they fit with the 1/f model, we confirmed that realistic noise commonly deviated from the 1/f model to some degree (*p* = 0.02~0.07, F-test). Therefore, different frequency bands possessed unique advantages when confronted with complex noise conditions.

Based on the aforementioned analysis, integrated waveforms from multiple filters could provide more information about the original spike waveform, which may pave a new way for a high-performance spike sorting algorithm. In the common spike sorting pipeline, a single high-pass filter with an empirical cut-off frequency at 300 Hz is used to remove the slower oscillations. In this study, in order to effectively leverage the effects of different cut-off frequencies on the waveform shapes, we applied multiple high-pass filters to process the raw data. The combination of multiple filtered data held the potential to enhance the sorting performance.

### 2.2. Multi-Frequency Filtering

Multi-frequency filtering is accomplished by using several forth-order high-pass Butterworth filters with different cut-off frequencies. For the neural signal collected by electrodes, we chose 3 high-pass filters with low, medium, and high levels of cut-off frequencies to filter the signal, respectively, as shown in Formula (1):

(1) $$\begin{array}{c} D_{l}=Butterworth\left(D,w_{l},a\right)\\ D_{m}=Butterworth\left(D,w_{m},a\right)\\ D_{h}=Butterworth\left(D,w_{h},a\right) \end{array}$$

where $D_{l}$,$\;D_{m}$, and $D_{h}$ denote the filtered signal with low, medium, and high levels of cut-off frequencies, respectively. $Butterworth\left(\cdot \right)$ indicates Butterworth filter expression; D is the raw signal; and $w_{l}$, $w_{m}$, and $w_{h}$ are the low, medium, and high levels of frequency bands, respectively. a is the filter order, which was set to a=4.

### 2.3. Composite Spike Waveform Detection

For the neural signal $D_{l}$, filtered with a low-frequency band, the spikes were detected by thresholding. For instance, the double thresholding of Formula (2):

(2) $$Thr=\pm 4\times median\left\{\left|D_{l}\right|/0.6745\right\}$$

For each detected spike, L points were cut for further analysis. Their peaks were identified, $P=\left\{P_{1},P_{2},\ldots ,P_{N}\right\}$, and then aligned to P at the kth data point, as shown in Formula (3):

(3) $$S_{l}=\left[D_{l}^{P-k+1},\ldots ,D_{l}^{P},\ldots ,D_{l}^{P+L-k}\right]$$

$S_{l}$ denotes the spikes detected from $D_{l}$; $D_{l}^{i}$ is the ith points in $D_{l}$; and $D_{l}^{P}$ denotes the peaks located at the kth point.

For $D_{m}$ and $D_{h}$, the spikes were aligned at the peaks index P obtained from $D_{l}$, and then the L points were cut:

(4) $$S_{m}=\left[D_{m}^{P-k+1},\ldots ,D_{m}^{P},\ldots ,D_{m}^{P+L-k}\right]$$

(5) $$S_{h}=\left[D_{h}^{P-k+1},\ldots ,D_{h}^{P},\ldots ,D_{h}^{P+L-k}\right]$$

$S_{m}$ and $S_{h}$, respectively, denote the spikes cut from $D_{m}$ and $D_{h}$; $D_{m}^{P-k+1}$ and $D_{m}^{P+L-k}$, respectively, denote the $\left(P-k+1\right)\mathrm{th}$ and (P+L−k)th points in $D_{m}$, and so on. $D_{m}^{P}$ denotes the value in $D_{m}$ at the peak of $D_{l}$, located at the kth point, and so on as $D_{h}^{P}$.

Finally, we spliced the generated spike waveforms of low-, medium-, and high-frequency bands to generate the composite spike waveform:

(6) $$S_{MultiFq}=\left[S_{l},S_{m},S_{h}\right]$$

$S_{MultiFq}$ denotes the composite spike waveform from multiple frequency bands.

### 2.4. Feature Extraction

This study mainly employed Principal Component Analysis (PCA) for feature extraction [24]. PCA is a widely used linear dimensionality reduction method with an outstanding advantage of simplicity. In order to retain as much information as possible, PCA takes the directions with the largest variance as the projection directions of data. To illustrate, the direction with the largest variance is taken as the first coordinate axis of the projection subspace, and the direction that is orthogonal to the first axis with the second largest variance is taken as the second coordinate axis, and so on, to construct the subspace. It has been reported by most of the previous studies [25] that a 3D PCA-subspace is enough to provide discriminative features for clustering. Therefore, in this study, we extracted the first three components of PCA, allowing the data to be projected into a 3D subspace to extract relevant features.

### 2.5. Clustering

K-means clustering is one of the simplest clustering algorithms [26]. The procedure is to select K points as the initial cluster centers randomly. For each data point, calculate its distance to each cluster center and then assign it to its nearest cluster center. As a data point is assigned, recalculate the cluster center according to the current points in the cluster. The above process is repeated until there are no further changes in the cluster centers or points assignment.

### 2.6. Evaluation

#### 2.6.1. Datasets

We chose one simulated dataset and one in vivo dataset for performance evaluation. The simulated dataset Wave_clus  provided by Quiroga et al. [6] was used in this study. To date, Wave_clus  has been used by many spike sorting algorithms for evaluating sorting performance [27,28,29]. In the Wave_clus dataset, the inter-spike intervals of spike waveforms follow a Poisson distribution, and the noise exhibits a similar power spectrum to those of spikes. In addition, actual conditions such as spike overlap, electrode drift, and explosive discharge are simulated. Wave_clus contains four sets of data, C1, C2, C3, and C4. Each testing set contains three distinct spike waveform templates with varying levels of template similarity (C2, C3, and C4 > C1), and the background noise levels are quantified in terms of their standard deviations: 0.05, 0.10, 0.15, 0.20 (C1, C2, C3, and C4), 0.25, 0.30, 0.35, and 0.40 (C1). The waveforms detected from the Wave_clus dataset last about 2.5 ms and comprise 64 sample points. The peak values are aligned at the 20th sample point. Thus, the composite waveforms from three levels of frequency bands have 192 sample points.

The in vivo extracellular recordings can compensate for some shortcomings of the simulated data. These recordings capture the variability inherent in spike waveforms, which is lacking in simulations. Here, we chose a publicly available in vivo dataset HC1, which contained the extracellular and intracellular signals from rat hippocampal neurons with silicon probes [30]. We used the synchronized intracellular recording as the label information of extracellular recording to obtain partial ground truth [30]. If the difference between the extracellular spike time and the intracellular peak time was less than 0.3 ms, it was regarded that the spikes came from the same action potential.

We used six testing sets, d533101, d1122206, d681106, d1282103, d1352202, and d1512101, in HC1 for feasibility illustration, with 4 testing sets, d533101, d1122206, d1282103, and d1512101, for performance evaluation. Taking d533101 as an example, it contained the intracellular potential of a single neuron and the simultaneous extracellular waveforms of this single neuron along with some other neurons. We detected 3000 extracellular spikes from extracellular recording and 849 intracellular action potentials from intracellular recording. After analysis, 800 spikes in the extracellular recording corresponding to the action potentials in the intracellular recording were used as ground truth. We will refer to them later as marked spikes; the remaining 2200 spikes were unmarked. The waveforms detected from the HC1 were composed of 32 sample points. The peak values were aligned at the 11th sample point. Thus, the composite waveforms from three high-pass filters had 96 sample points.

#### 2.6.2. Performance Measure Metrics

One of the performance measure metrics was sorting accuracy, which was the percentage of the detected spikes labeled correctly. For sample set D, the accuracy of classification algorithm f was defined as the ratio of the number of spikes correctly classified to the total number of spikes used for classification, as shown in Formula (7):

(7) $$Accuracy\left(f,D\right)=\frac{1}{n}\sum_{i=1}^{n}\left(f\left(x_{i}\right)=y_{i}\right)$$

$x_{i}$ denotes the detected spikes, $y_{i}$ denotes the labeled spikes for ground truth, and n denotes the total number of spikes.

Another metric is the inter-class separation, which measures the clustering quality as is shown in Formula (8), where a larger separation value indicates a higher quality of clustering.

(8) $$SP_{ab}=|\left|y_{ca}-y_{cb}\right|{|}_{2}$$

$SP_{ab}$ denotes the inter-class distance between the clusters $C_{a}$, $C_{b}$, and $y_{ca}$, and $y_{cb}$ denotes the center of the cluster $C_{a}$ and $C_{b}$, respectively.

To evaluate algorithm performance on real dataset HC1 with partial ground truth [30], we solved it as a binary classification problem. The classification results were divided into four cases: True Positive (TP), False Positive (FP), True Negative (TN), and False Negative (FN). We evaluated the performance of the algorithm in terms of three indicators: Precision, Recall, and F1 score [31], as shown in Formulas (9)–(11).

(9) Precision=1−False Positive Rate

(10) Recall=1−False Negative Rate

(11) $$F1=2*\left(Precision*Recall\right)/\left(Precision+Recall\right)$$

We also adopted a time consumption measurement, running time, to investigate the time complexities of methods and their capabilities to real-time online applications.

## 3. Results

**Figure 2:**
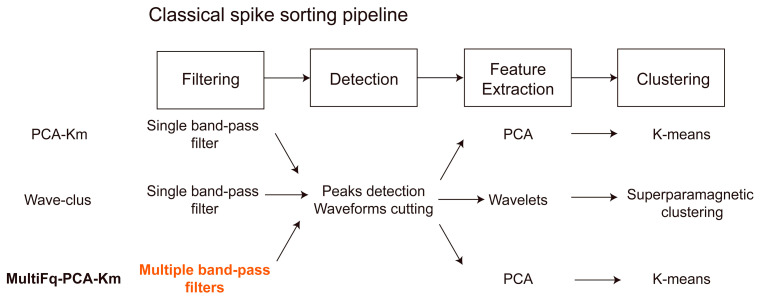
The algorithms used for each processing step of PCA-Km, Wave-clus, and MultiFq-PCA-Km. Our proposed method applies multiple band-pass filter in the filtering process, which is marked with orange color.

To illustrate the effective improvement of the proposed method MultiFq, we combined it with a simple and classic spike sorting method, PCA-Km [32]. Then we compared the performance between the newly generated MultiFq-PCA-Km and the original PCA-Km, along with a widely used spike sorting toolbox Wave-clus [11,32], on one simulated dataset and one in vivo dataset (Figure 2). Finally, to validate the generalizability of our method, we further combined the proposed MultiFq with two other spike sorting algorithms, Wave-clus and LPP-LSC [33], and presented the performance improvements on simulated datasets. All the results were performed using an Intel (R) Core (TM) i7-7700 central processing unit (CPU) equipped with 16 GB random access memory (RAM) without GPU-accelerated processing.

### 3.1. Performance Evaluation with the Simulated Dataset

**Figure 3:**
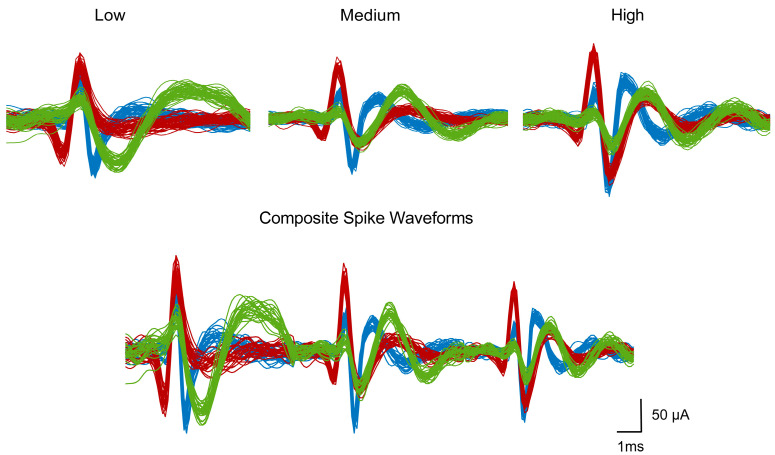
An example of the generated spikes after filtering with three levels of frequency on dataset C1_005. The Low, Medium, and High panels correspond to low (300–6000 Hz), medium (700–6000 Hz), and high (1000–6000 Hz) levels of frequency, respectively. The Composite Spike Waveforms panel corresponds to the spliced waveforms of all three frequency bands. The spike waveforms are colored according to the ground truth, in which red, green, and blue represent cluster 1, cluster 2, and cluster 3, respectively.

We first examined the performance of the three algorithms (MultiFq-PCA-Km, PCA-Km, and Wave-clus) on the simulated dataset Wave_clus, excluding the overlapping spikes. To compare the performance of each algorithm, three metrics were employed in this study: sorting accuracy, inter-class separation, and running time. The Wave_clus was sampled at 25 kHz, and we set the filter parameter $w_{l}=300–6000\;\mathrm{Hz}$, which was the most commonly used filtering frequency band [3,34,35] in previous studies, along with two higher frequency bands, $w_{m}=700–6000\;\mathrm{Hz}$ and $w_{h}=1000–6000\;\mathrm{Hz}$, i.e., the signal was filtered between three levels of frequency bands: 300–6000 Hz, 700–6000 Hz, and 1000–6000 Hz. After multi-frequency filtering, spikes were detected and aligned for each frequency band. Figure 3 is an example of the generated spikes on dataset C1_005, where the Low, Medium, and High panels correspond to three levels of filtering frequency, respectively. By splicing three of them, and the composite spike waveforms from multiple frequency bands are generated for the following sorting, as shown in the Composite Spike Waveforms panel in Figure 3.

To intuitively compare the performance of each algorithm concerning different noise levels, we plotted the accuracy, separation, and running time on four simulated datasets (C1, C2, C3, and C4). The dataset C1 contained eight testing sets, whereas C2, C3, and C4 all contained four testing sets. In each dataset, the noise levels of testing datasets gradually increased.

**Figure 4:**
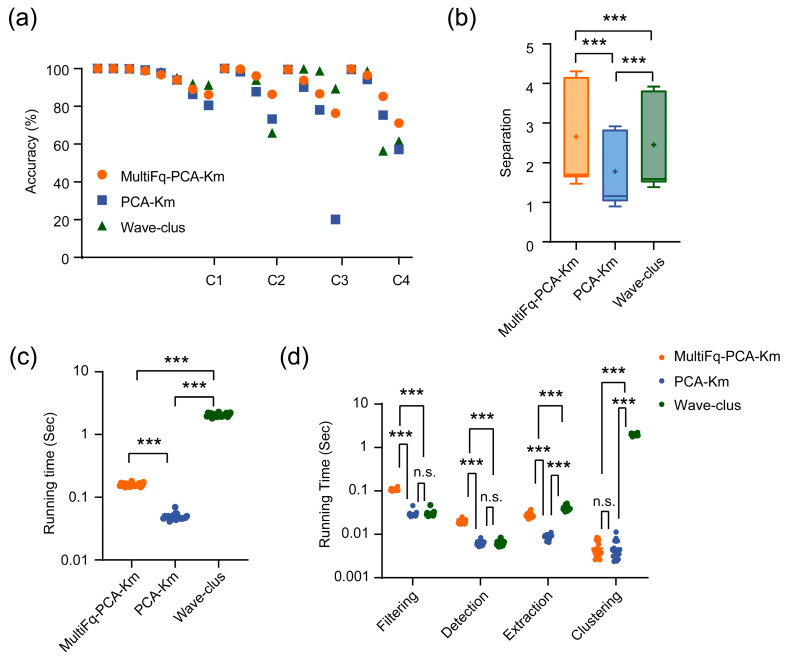
The performance comparison among three spike sorting algorithms on simulated dataset Wave_clus. (**a**) The sorting accuracy of three algorithms on all 20 testing sets with different noise levels. (**b**) Comparison of the inter-class separation on all 20 testing sets. *** *p* < 0.001, Wilcoxon matched-pairs signed rank test. The plus signs represent the mean value. (**c**) Comparison of the running time on all 20 testing sets; the solid lines represent the mean value. *** *p* < 0.001, Paired-*t* test. (**d**) Comparison of the running time for each processing step on all 20 testing sets. *** *p* < 0.001, n.s. represents no significance, Paired-*t* test.

Among all 20 testing sets, the MultiFq-PCA-Km achieved the highest average accuracy up to 92.8%, whereas the original PCA-Km was 86.6%, and Wave-clus was 92.0%. Figure 4a shows the sorting accuracy comparison between these three algorithms. It is worth noting that the proposed MultiFq when combined with PCA-Km outperformed the original PCA-Km and the widely used toolbox Wave-clus. Especially for those testing sets near the end of each dataset, where the noise level was relatively higher, the accuracy gap between the MultiFq-PCA-Km and PCA-Km was more evident. In other words, when the spike sorting difficulty increased, MultiFq brought more robustness to the sorting method. Another metric, inter-class separation, confirmed the above conclusion. As shown in Figure 4b, MultiFq-PCA-Km presented a significantly larger separation compared to PCA-Km, which meant the higher quality of clusters produced by MultiFq-PCA-Km (*** *p* < 0.001, Wilcoxon matched-pairs signed rank test). Moreover, the separation of MultiFq-PCA-Km was also significantly higher than Wave-clus (*** *p* < 0.001, Wilcoxon matched-pairs signed rank test).

While improving the sorting performance, MultiFq-PCA-Km did not increase much computational time. Figure 4c presents the advantages of MultiFq-PCA-Km on running time. The mean running times of MultiFq-PCA-Km, PCA-Km, and Wave-clus were 0.16 ± 0.01 s, 0.05 ± 0.00 s, and 2.06 ± 0.13 s, respectively. The introduction of the MultiFq did not add much additional time consumption compared to PCA-Km. And, MultiFq-PCA-Km was more than ten times faster than Wave-clus. We further analyzed the time consumption of each processing step in the different algorithms (Figure 4d). The results indicated that the additional time was mainly used for filtering raw data by multiple filters and detection from the filtered data in more dimensions.

It was undeniable that the accuracy of MultiFq-PCA-Km was slightly inferior to Wave-clus on some of the testing sets in C3. However, this minor defect was acceptable, given the time-consuming nature of Wave-clus. MultiFq significantly reduced the running time of the whole algorithm (Figure 4c, *** *p* < 0.001, Wilcoxon matched-pairs signed rank test). Wave-clus finished sorting in seconds, whereas MultiFq-PCA-Km ran at the millisecond level. In summary, MultiFq achieved considerable optimization in sorting performance and time consumption.

### 3.2. Performance Evaluation with In Vivo Dataset

We further evaluated the performance of MultiFq-PCA-Km and the other two algorithms on four testing sets extracted from the in vivo dataset HC1: d533101, d1122206, d1282103, and d1512101. HC1 was sampled at 10 kHz. We set the filter parameters $w_{l}=300–3000\;\mathrm{Hz}$, $w_{m}=500–3000\;\mathrm{Hz}$, and $w_{h}=700–3000\;\mathrm{Hz}$. The filter upper limit was set according to the sampling rate [7,36,37,38]. The testing sets contained marked spikes and unmarked spikes from rat hippocampal neurons, in which the marked spikes corresponded to intracellular potential and belonged to the same cluster, whereas the cluster labels of the unmarked spikes were unknown. According to the above partial ground truth, we analyzed four metrics: Precision, Recall, F1 score, and running time of each algorithm.

**Figure 5:**
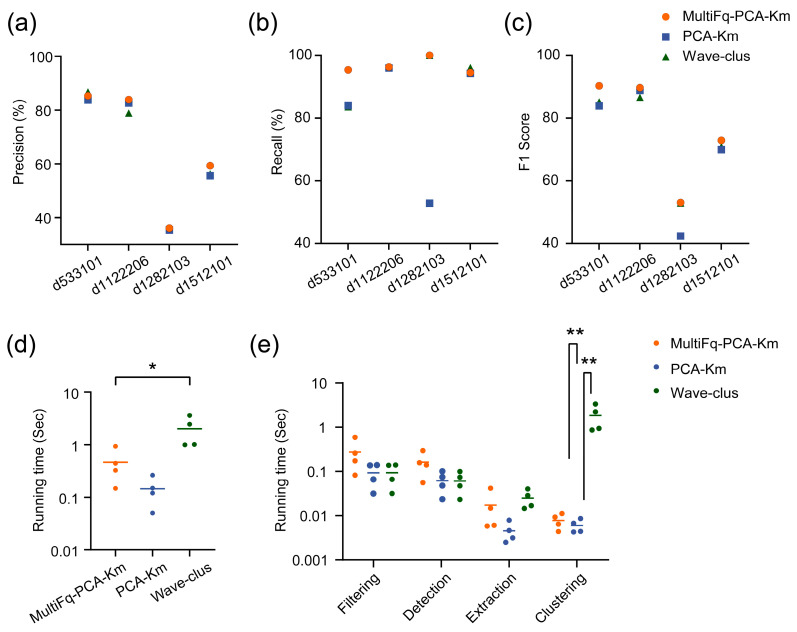
The sorting performance metric comparison among three spike sorting algorithms on in vivo dataset HC1. (**a**) Precision. (**b**) Recall. (**c**) F1 score. (**d**) Running time. The solid lines represent the mean value. * *p* < 0.05, Paired-*t* test. (**e**) Comparison of the running time for each processing step on in vivo dataset HC1. ** *p* < 0.01, Wilcoxon matched-pairs signed rank test. Only the significant differences are marked in the figure.

A comparison of each algorithm is shown in Figure 5. Figure 5a,b shows a higher Precision and Recall of the MultiFq-PCA-Km than PCA-Km. F1 score, as a comprehensive measurement, is the harmonic mean of Precision and Recall. In Figure 5c, the F1 score of MultiFq-PCA-Km is evidently higher than the original PCA-Km, indicating substantial advantages of combining the proposed MultiFq and PCA-Km. We also noticed that there was little difference between the sorting performance of MultiFq-PCA-Km and Wave-clus. Considering that Wave-clus is a time-consuming algorithm with high time and computation complexity, the running time measurement can show the advantage of our proposed method. We performed the same analysis of time measurement for the whole algorithm and each processing step as before (Figure 5d,e). The mean running times of MultiFq-PCA-Km and PCA-Km were 0.46 ± 0.34 s and 0.17 ± 0.01 s, respectively. Notably, introducing the MultiFq brought only a little additional time (no significance, Paired-*t* test) for the process. On the contrary, the running time of Wave-clus was up to 3.63 s and significantly higher than that of our proposed method (* *p* < 0.05, Paired-*t* test). All the above results indicated that our proposed method could be applied to online spike sorting with an outstanding balance between sorting performance and computational time consumption.

### 3.3. Compatiblity of the Proposed MultiFq

**Figure 6:**
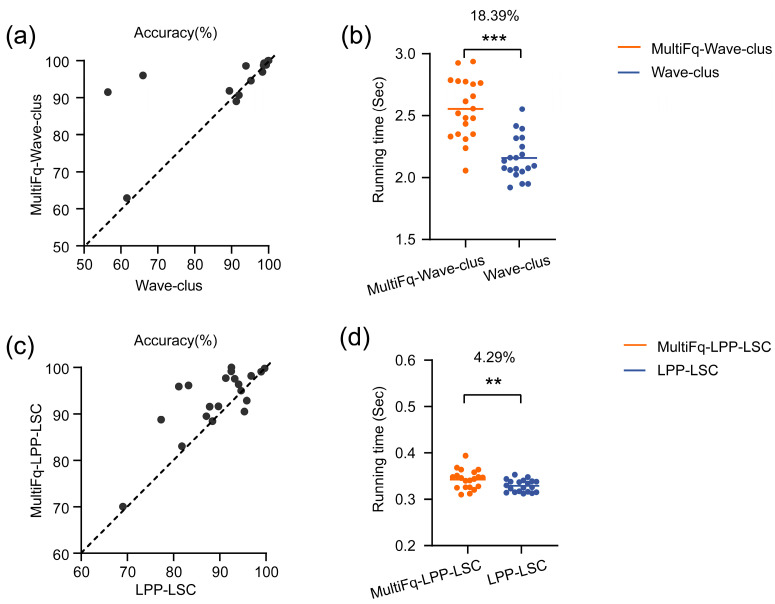
The performance evaluation on two algorithms, Wave-clus and LPP-LSC, on simulated dataset Wave_clus. (**a**) The sorting accuracy of MultiFq-Wave-clus and the original Wave-clus on all 20 testing sets with different noise levels. (**b**) Comparison of the running time between MultiFq-Wave-clus and the original Wave-clus on all 20 testing sets; the solid lines represent the mean value. *** *p* < 0.01, Paired-*t* test. (**c**) The sorting accuracy of MultiFq-LPP-LSC and the original LPP-LSC on all 20 testing sets with different noise levels. (**d**) Comparison of the running time between MultiFq-LPP-LSC and the original LPP-LSC on all 20 testing sets; the solid lines represent the mean value. ** *p* < 0.01, Paired-*t* test.

The proposed method MultiFq could be combined with various existing spike sorting methods and perform well, regardless of how the sorting methods work. To verify this, we chose two widely used algorithms Wave-clus and LPP-LSC [25,39]. Wave-clus used wavelet transform to extract features and SPC to do clustering, whereas LPP-LSC used locality preserving projection (LPP), one of the dimension reduction methods, to extract features and landmark-based spectral clustering (LSC) to do clustering. Then we tested the performance of two more combinations, MultiFq-Wave-clus and MultiFq-LPP-LSC, on the dataset Wave_clus. The comparison results with the original algorithms are presented in Figure 6.

The Wave-clus algorithm contains some key parameters that affect the number of clusters in sorting results. Thus, to exclude the influence of errors in the number of clusters on the accuracy, we set the parameters uniformly for the dataset so that the algorithm could correctly identify the number of clusters. Figure 6a shows that the combination of MultiFq and Wave-clus presents a higher level of sorting accuracy. It was noticed that the accuracies of the two datasets were improved from 56.44% to 91.48% and from 81.14% to 95.88%, respectively.

As shown in Figure 6c, MultiFq-LPP-LSC outperformed the original LPP-LSC since its accuracy was higher on 17 out of all 20 testing sets (17 points above the diagonal). Also, the average accuracy of MultiFq-LPP-LSC across all testing sets reached 92.3%, whereas it was only 90.1% for the LPP-LSC.

It is also worth noting that no points under the diagonal were found far away from the diagonal, whereas many points above and the diagonal were found far away from the diagonal, which mainly represented the results from the datasets with higher noise levels. We found that the maximum improvements brought by combining our MultiFq were 35.04% in Wave-clus and 14.74% in LPP-LSC in our testing datasets. All of these results confirmed the robustness of our proposed method once again.

Similar to the analysis in the above section, we performed the running time measurement of these original and combined algorithms. As shown in Figure 6b,d, MultiFq-Wave-clus and MultiFq-LPP-LSC increased the time consumption by 18.39% (0.4 s) and 4.29% (0.014 s), respectively (*** *p* < 0.001, ** *p* < 0.01, Paired-*t* test). This meant the combination of MultiFq and the sorting methods only brought a little additional time consumption but an improvement in sorting performance.

## 4. Discussion

In this study, we modified the filtering process of the common spike sorting with three parallel high-pass filters to generate composite waveform shapes for optimizing spike sorting. By combining waveform shapes from three frequency filters, we obtained more informative features compared to the single waveform shape obtained from a conventional filter. Our evaluation, conducted on both a simulated- and in vivo dataset demonstrated that the proposed MultiFq could improve the sorting performance with little increase in computational consumption. Our results also demonstrated that the proposed method interfaced well with various sorting methods, which indicated that it was compatible with any spike sorting algorithm following the “Spike Detection—Feature Extraction—Clustering” pipeline.

Different from the optimization of feature extraction and back-end clustering steps in most of the previous studies of spike sorting methods, our proposed MultiFq optimized the filtering process, which aimed to leverage more information from original waveforms prior to spike detection. The principle of spike sorting was based on distinguishing different waveform features. Compared to the spike sorting methods based on a single filter, our MultiFq spike sorting method outperformed them by utilizing composite waveform shapes. By leveraging the features captured by multiple high-pass filters, the composite waveform shape was able to make more use of the information contained in the raw neural signals and obtain evident improvements in performance with little extra computing burden.

Our MultiFq was competitive with the state-of-the-art algorithms, especially for online spike sorting, due to the balance between computational complexity and sorting performance. Several applications, like brain machine interfaces or closed-loop experiments [40], will require accurate online spike sorting. Although some deep learning methods, like 1D-CNN [41,42], have shown high accuracy, they inevitably bring high computation complexity. Additionally, deep learning methods need training data with ground truth, which is difficult for in vivo spike sorting. Another type of automatic unsupervised algorithm, like LDA-Gmm or LDA-DP [23,25], requires numerous iterations and a long operation time, which is also unsuitable for online applications. By contrast, our results have demonstrated that the proposed method, which was mainly used for the multiple-filtering process, only brought a little additional time consumption while improving sorting performance. And, the extra time brought by multiple filters in our MultiFq was much less than that brought by clustering in Wave-clus (Figure 4c and Figure 5e). In principle, multiple filters could be carried out through parallel calculation and hardware acceleration, such as with parallel GPUs, and could be easily implemented for further improving performance. Therefore, our method could be run “in time”. In addition, multiple parallel filtering processing is much easier implemented in hardware than complex clustering processes, such as SPC. At the same time, the low computation burden is also essential for miniaturized hardware implementations [43], which gives it advantages over complex approaches to online processing.

Since our method is an improvement in filtering process, it can be fully integrated into various existing spike sorting methods. As our results demonstrated, when MultiFq was applied to three typical spike sorting algorithms, including PCA-Km, Wave-clus, and LPP-LSC, considerable improvements were achieved in the combination algorithms with low time consumption compared with the original ones. It is worth noting that when our proposed method combined with Wave-clus, MultiFq-Wave-clus increased time consumption by 18.39%, whereas MultiFq- LPP-LSC brought only a 4.29% time increase. The possible reason for this was that Wave-clus applies a wavelet transform before dimension reduction in the feature extraction step. The composite spike waveforms contain three times more sample points than the original waveform. Although dimension reduction is employed in the feature extraction step of both Wave-clus and LPP-LSC, the wavelet transformation processing before dimension reduction made Wave-clus spend much more additional consumption time than LPP-LSC.

In addition to this, over the last decades, the development of high-density microelectrode arrays, such as Neuropixels, has allowed an increase in the number of recorded neurons. The newly developed spike sorting methods, like MountainSort and KiloSort, combine the temporal and spatial information obtained from the high-density microelectrode arrays and achieve high performance in spike sorting [44,45]. In theory, our proposed method is also beneficial for these new spike sorting methods in the temporal domain with a high-density microelectrode array. However, in this study, we only evaluated the performance of our proposed method and the combined methods with the in vivo dataset obtained from the conventional sparse microelectrode array. Therefore, whether or not the combination of our proposed MultiFq with the newly developed spike sorting methods would bring a performance improvement in spike sorting with a high-density microelectrode remains to be further explored.

## 5. Conclusions

Accurate spike sorting to the appropriate neuron is crucial for neuroscience and neurotechnology studies. Many advanced spike sorting methods have been developed over the past decades. However, unlike feature extraction and clustering, the first filtering step in the spike sorting has been largely neglected in previous studies. In this study, we modified the filtering process with three parallel high-pass filters to generate composite waveform shapes designed to capture more time-domain features of spike waveforms during both depolarization and hyperpolarization. Our results from the simulated and in vivo datasets with ground truth demonstrated that the proposed MultiFq dramatically increased the accuracy of spike sorting and saved the consumption time compared to the classical sorting methods. We also demonstrated its good compatibility with existing sorting methods, with significant performance improvements and little additional computational costs. Our promising results indicated that optimizing the filtering step would be an alternative way to improve the performance of spike sorting algorithms.

## Data Availability

The datasets and codes for this study are available from the corresponding authors upon reasonable request.
